# Real-World Views of Patching Differ to Health Professionals’: An Online Survey of Professionals, Patients, Teachers, Parents and Carers

**DOI:** 10.22599/bioj.404

**Published:** 2025-04-29

**Authors:** Daniel Osborne, Maddison McGowen, Jeremy Bradshaw, Helen Ellis, Megan Evans, James Stallwood, Joerg Fliege, Jay Self

**Affiliations:** 1 University Hospital Southampton NHS Foundation Trust, Southampton, UK; 2 Faculty of Medicine, University of Southampton, Southampton, UK; 3 Faculty of Social Sciences, University of Southampton, Southampton, UK

**Keywords:** Amblyopia, Occlusion therapy, Eye patch, Survey

## Abstract

**Background:**

Patching therapy is the most common treatment for amblyopia (lazy eye) and is unsuccessful for approximately 40% of patients, leaving them with life-long unilateral visual impairment and increased risk of bilateral visual impairment later in life. Poor adherence to patching therapy is a major contributing factor in treatment failure yet we lack real-world understanding as to why this is a problem outside of controlled research studies.

**Methods:**

In collaboration with patient contributors, we developed an online survey for past patients, parents/carers of children with amblyopia, health professionals, and schoolteachers. The survey included questions about when and where is best for children to wear the patch, the design of the patch, and facilitators and barriers to patching therapy.

**Results:**

We received 631 responses to the survey (259 health professionals, 213 parents/carers, 110 people who patched as a child, 7 teachers, and 42 people matched to multiple categories). Healthcare professionals thought weekday (54.4% versus 14.3% preferring weekend and 31.3% no difference) and school (54.4% versus 21.6% preferred home and 23.9% no difference) patching was more successful. Past patients (52.4%) favoured ‘force’ as a technique to encourage patching; more than both health professionals (7.7%) and parents or carers (19.7%). Patients rated ‘people making fun’ of them as an important barrier to patching.

**Conclusions:**

We describe surprising differences in stakeholders’ responses to the survey questions about barriers to successful patching treatment. We suggest these differences are used as a guide for further work to explore stakeholder’s social experience of patching.

## Introduction

Amblyopia (lazy eye) is a cortical visual impairment characterised clinically by unilateral or bilateral reduced visual acuity in the absence of explanatory structural eye abnormalities. It is the main cause of preventable childhood visual impairment (prevalence = 1.0 to 5.5%), and a common cause of unilateral visual impairment in adults (Attebo *et al.*, 1998; Cruz *et al.*, 2023). The condition affects males and females equally, is more common in children born prematurely or with low birthweight for gestation age, have a first degree relative with amblyopia, or have a neurodevelopmental condition (Williams *et al.*, 2008). Environmental risk factors include maternal smoking, substance and/or alcohol use in pregnancy (Williams *et al.*, 2008). Caused by of loss of vision through injury or disease to their fellow (‘normal’) eye, people with amblyopia have increased risk of bilateral visual impairment over their lifetime (Rahi *et al.*, 2002).

Patching therapy has been the mainstay of treatment for amblyopia for many decades (Cruz *et al.*, 2023). However, forty percent (40%) of children have a greater than two-line inter-ocular visual acuity (VA) difference at the end of therapy (Awan *et al.*, 2010). Results from invasive animal model studies (Kiorpes, 2019), and randomised controlled trials (RCTs) of interventions for amblyopia (Scheiman *et al.*, 2005), suggest there is a critical period for the successful management of amblyopia between ages 0 and approximately 7 to 12 years. Current practice in the UK (and similar in many developed countries worldwide) is to identify cases using a population screening programme at age 4–5 years (Solebo, Cumberland and Rahi, 2015), leaving 2–3 years for amblyopia management before the earliest proposed end of the critical period of visual development.

The literature on why so many children do not achieve a less than two-line interocular VA difference within this period is inconclusive. The most persuasive hypothesis is that poor outcomes are driven by children unable to adhere to best practice therapies, which, unchanged since Victorian times, involve correction of refractive error with spectacles followed by patching therapy of the better seeing eye for 2 to 6 hours per day for 18 months (Awan *et al.*, 2010).

The timing and duration of patching has been the focus of many research studies and is still under scrutiny. For example, a recent large RCT that showed the benefits of early patching versus full optical correction with 33% of children in the ‘early patching’ group not achieving a less-than-two-line interocular VA difference during the 24-week trial follow-up period (Proudlock *et al.*, 2024). Heat sensitive dose monitor studies have found that many children do not complete the full dose of therapy, and those that don’t are most likely to have an unfavourable outcome (Stewart *et al.*, 2007, 2017). Recent advances in binocular stimulation therapies have not been shown to be superior to patching therapy (Pineles *et al.*, 2020; Tailor *et al.*, 2022; Tsani *et al.*, 2024) and, with reliance on expensive technology, will likely increase health inequality. Additionally, they limit the activities that children can undertake whilst having the therapy.

It seems likely that a key component of improving outcomes for the population of people with amblyopia is to improve adherence to current best practice. Dean *et al*. (2016), published a review of nine studies examining interventions to improve adherence to patching therapy. They found educational information for parents and carers appeared most effective at improving self-reported adherence to patching. Amending the patching protocol (for example, by recommending breaking the patching dose into two session per day) and forcing children to wear the patch (e.g. forcibly fixing the patch to the face with glue) did not appear effective. In the time since this review there have been theoretical and technical advances that could potentially assist in addressing the problem of adherence to patching therapy. Firstly, methods to involve children and stakeholders in the development of healthcare interventions (for example, the Person-Based Approach) (Yardley, Ainsworth, *et al.*, 2015; Yardley, Morrison, *et al.*, 2015)) provide a framework to identify what is important to various stakeholders and synthesise their values into practical solutions to clinical problems. Secondly, wide access to internet, smartphones and communication technology provides platforms to communicate with patients and develop engaging education interventions that was previously not possible.

Here, we use an easy access online survey of all stakeholders to gain a greater understanding of their experience of patching therapy in the real-world outside of a clinical study environment. There are multiple stakeholders and groups involved in a child’s patching therapy, each with a different lens and experience of patching. In this study we explore the reported barriers to and facilitators of adherence with patching therapy. We then, for the first time, explore the differences in these opinions between different stakeholder groups. A theoretical model of what is important to each stakeholder group could inform clinicians’ management of patients and their families; guide policy; support evidence for the research and development of novel interventions exploiting advances in technology; and inform the development of complex healthcare interventions.

## Methods

The study was reviewed by Leicester South NHS Research Ethics Committee (reference number: 16/EM/0418). The study did not require participants to give written informed consent because it did not collect personal identifiable data.

We developed a questionnaire in collaboration with patients, their parents/carers, and stakeholders before administering it to various professionals, patients and families online. Our research team, comprising UK clinical professionals (orthoptists and ophthalmologists) completed a search of the literature to identify facilitators and barriers to patching therapy adherence. Using our clinical expertise and findings from the literature review, we developed a first draft of the questionnaire. We held meetings with members of our local Patient and Public Involvement and Engagement (PPIE) groups including eight parents and their six children between ages 5 and 18 years, five clinicians and a patient group advocate to refine the content (themes) of the questionnaires and the wording of individual questions. Changes to the questionnaire through our PPIE work included:

a) introduction of additional branching to tailor the questionnaire based on the respondents’ previous answers,
b) re-wording of some questions into more widely understood (lay) language, and
c) removal of some questions to reduce the time taken to complete the questionnaire.

The final questionnaire asked each participant group (parents/carers, teachers, previous patients, and current patients) questions under the following themes:

- When and where is it best to wear the patch?
- Which type of patch is best to wear?
- What are the barriers and facilitators to wearing the patch?

Questions were worded differently for each stakeholder group using a branching method and according to expected reading ability (full list of questions available in Tables S1 to S5). The questionnaire was advertised online through broad social media network dissemination, by professional body online and print communications, through patient support charities, and clinician and research professionals through social media and various online web resources including a national email listserv platform. We use descriptive analysis to present participant’s responses.

## Results

### Demographics

**Figure 1:**
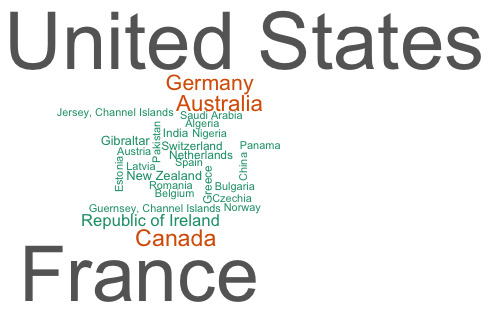
Word cloud: frequency of respondent’s country of residence excluding United Kingdom. A larger word indicates greater frequency.

We had 631 responses to the survey comprising 259 health professionals, 213 parents/carers, 82 adults who patched as a child, 28 under 18s who had previously patched, 7 teachers, and 42 respondents who fitted into more than one category. Participants that fitted into multiple categories were excluded from the subgroup analysis. Most participants (n = 408, 65%) currently live in the UK with others reporting their home country as: United States of America (n = 74); France (n = 71); Canada (n = 14); Australia (n = 13); Germany (n = 12); Republic of Ireland (n = 7); and 23 other countries with 3 or fewer respondents (see Figure 1).

#### Where and when is it best to wear the patch?

**Figure 2:**
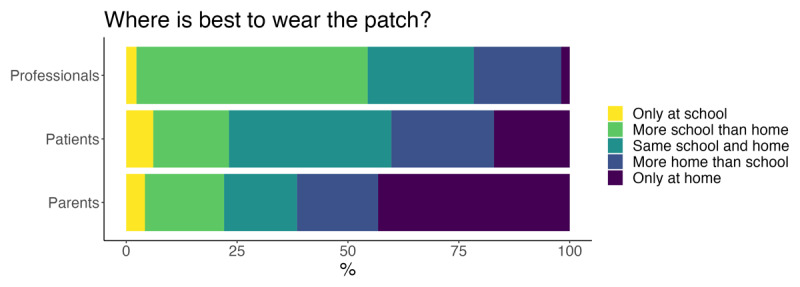
Where is patching is most successful?

Health professionals, past patients, and parents or carers were asked whether they thought patching at home or school was more successful (see Figure 2). Health professionals favoured school and weekend patching over home and weekday patching, whilst patients and parents/carers responses were more evenly spread with regard to where was most successful.

**Figure 3:**
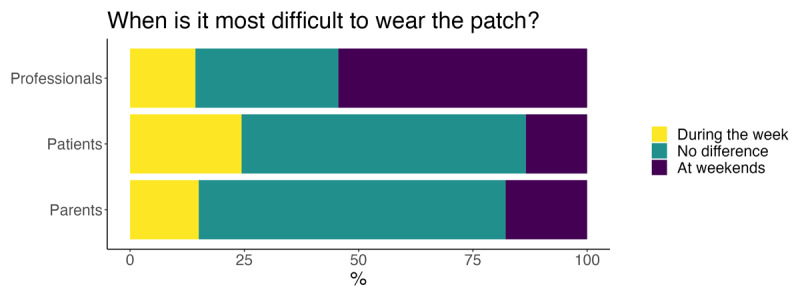
When is it most difficult to patch?

Participants were then asked if weekdays or weekends were a more difficult time to wear the patch. Shown graphically in Figure 3, previous patients and the parent/carer groups appeared to agree that there was no difference between weekday or weekend wear, while professionals favoured weekend patching.

#### Design of the patch

**Figure 4:**
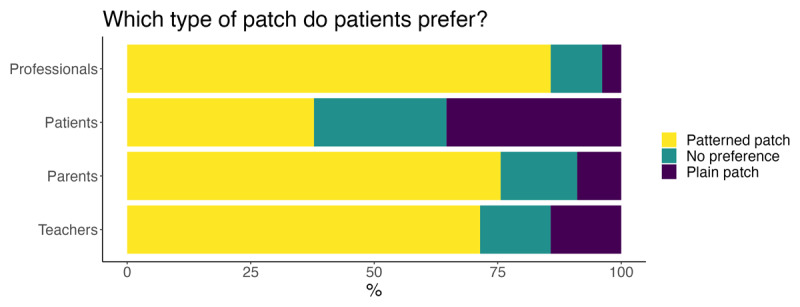
All groups preferred patterned patches, with the exception of past patients, the majority of whom either had no preference or preferred plain patches.

Teachers, parents/carers, past patients, and health professionals were asked about the design of patch. Which was preferable to children patching: plain or with a pattern. Most professionals (85.7%), parents (75.6%) and teachers (71.4%) felt that patterned patches were more appealing. Only 37.8% of past patients preferred this variety; 26.8% had no preference and 35.4% preferred plain patches (see Figure 4).

#### Facilitators and barriers to adherence of patching therapy

**Figure 5:**
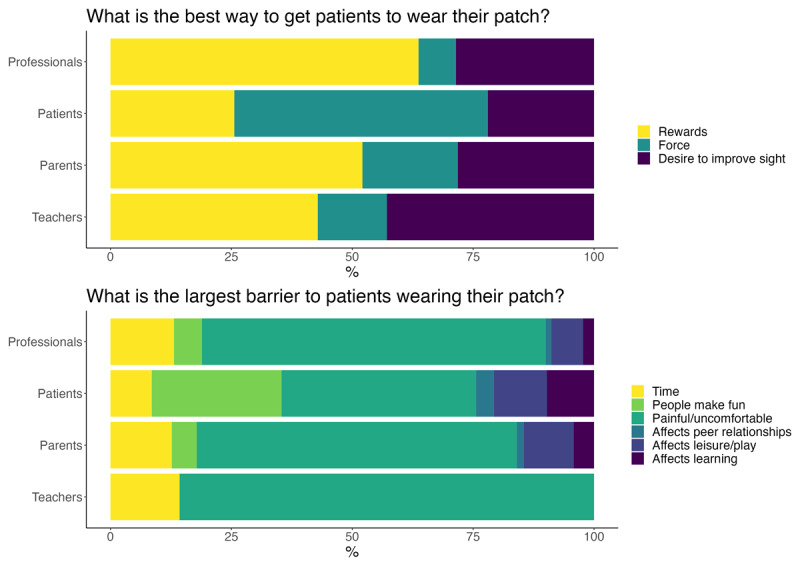
Past patients deviated in their responses to questions about barriers to patching therapy.

All participants were asked about facilitators and barriers to adherence to patching therapy. Given options of reward, force, or education on the importance of improved eyesight, most professionals and parents/carers preferred reward. Teachers placed equal value on reward and education. Again, past patients deviated from the other groups’ responses with the majority supporting ‘force’ rather than persuasion through education or reward. All groups recognised the practical barrier of time constraints on parents/carers and patients having time for the patching treatment. Professionals, parents/carers, and teachers felt that physical pain or discomfort caused by the patch was the most important barrier. Past patients responded more commonly that social factors, such as peers making fun of them, were more important (see Figure 5).

## Discussion

Our data has good representation from parents/carers, past patients, and eye healthcare professionals. We asked a range of questions that included how therapy could be improved and what barriers exist. The authors, comprising ophthalmic health professionals, academics, and a parent representative, were surprised to see observed differences between groups in their responses.

When asked when the patch should be worn (home versus school, weekday versus weekend), health professionals responded that school and weekdays are favourable. This contrasted with parents/carers and past patients, that preferred home and weekends. Clinicians likely feel sympathy for the families they see in clinic, who often tell of the challenges they experience at home. Our finding could be explained by clinicians’ sympathy for the families and their desire to move some of their strain onto school. Alternatively, clinicians, informed by the literature that emphasises the importance of the number of hours the patch is worn, attempt to encourage weekday patching because there is more scope for higher weekly doses.

Past patients’ responses to the type of patch favoured was also unexpected. While there was good agreement between professionals, parents/carers and teachers that patterned patches are better, fewer past patients preferred them. The survey cannot explain the reason for these responses, but we suggest that the design of the patch may matter more to an observer as they are the ones that primarily see it. The ‘observer’ is typically the person encouraging (read: making) the child wear the patch; a task they perceive as an inconvenience or trauma. The observer’s guilt over this may be partially alleviated if they see the child wearing an aesthetically pleasing patch. The design of the patch does offer opportunity to change the child’s narrative of patching therapy. For example, the child may be able to choose an animal they like or associate with comfort and reassurance. Or an intervention could develop a narrative of a character that wears and normalises the patch and appears both in educational material and on the patch design. It seems that further work is required that looks into more engaging and themed educational materials and patches.

Past patients again deviated from the other groups in their beliefs about barriers and facilitators to patching therapy. Surprisingly, past patients more commonly favoured ‘force’ over rewards. Force in the context of patching was not defined in our survey and could mean different things to different people. As has been seen in previous clinical trials, force could refer to a morally unacceptable super gluing of eye patches to children’s faces (Dean, Povey and Reeves, 2016). To a past patient ‘force’ could simply be a patient putting the patch on the child and distracting them with a toy. These are highly important differences that could be explored in qualitative work with past patients.

It is surprising that this group placed less value on rewards than the others and responded that social factors were greater barriers to adherence to therapy that other groups think. They placed greater importance on ‘people make fun of them’ as a barrier to patching therapy. This response perhaps is related to patients’ desire to patch more at home than in school. It suggests that part of the intervention to improve patching adherence could target education on patching therapy for the broader society.

Here, we have shown that different stakeholder groups have unexpected differences in their responses to questions about the most effective incentives and key barriers to adherence with patching therapy. We make some suggestions as to why these differences may exist and show that to address the problem of non-adherence, wider stakeholder input, beyond clinicians, is likely to be key. These findings could be used as a guide for the development of further research into patching therapy and the development of interventions to improve outcomes.

## Additional File

The additional file for this article can be found as follows:

10.22599/bioj.404.s1 Supplementary material. Tables S1 to S5.
