# Supplementary material for: Real-World Views of Patching Differ to Health Professionals’: An Online Survey of Professionals, Patients, Teachers, Parents and Carers
Source: Br Ir Orthopt J. 2025 Apr 29;21(1):59–65. doi: 10.22599/bioj.404 (PMC12047624; doi:10.22599/bioj.404)
Supplement: Supplementary material. — Tables S1 to S5. [file bioj-21-1-404-s1.pdf]

Supplementary material

Table S1: Under 18's questions

U18s

Are you currently based in the UK? Yes/no
We want to reach as many people as possible with this survey. To help us, what are the first 2 letters of your postcode

We want to reach as many people as possible with this survey. To help us, what country are you from? [Free text]

How old are you? [Free text number]

Which of the following applies to you? (I currently wear a patch / I used to wear a patch)

Feedback - Do you find it more difficult to patch during the week or at weekends? (During the week / No difference / Weekends)

I wear my patch: (Only at school / More school than home / Same at school and home / More home than school / Only at home)

Which type of patch do you prefer to wear? (Patterned patch / plain patch / No preference)
Overall, what makes you want to wear your patch the most? Please move the options and place in order. (Force / Rewards / Desire to improve eyesight)

Is there anything else that makes you want to wear your patch? [free text option]
In your experience, which of the following makes you not want to wear your patch? Time it takes / People make fun/ It's painful or uncomfortable / It affects my friendships / It changes how I play with my friends / It stops me from learning in school
Do you think that computer games or apps that could see if you are wearing your patch and give you extra skins/power-ups/lives/rewards would make you want to wear your patch? (Yes / no)

If so, how and why do you think this? [free text]
We are hoping to reach a wide range of voices with this survey, therefore we would like to know what is your highest educational attainment? (GCSE or equivalent, A-Level, Graduate degree)

Table S2: Questions to teachers

Teachers

Are you currently based in the UK? (Yes / no)

We want to reach as many people as possible with this survey. To help us, what are the first 2 letters of your postcode? [free text]

We want to reach as many people as possible with this survey. To help us, what country are you from? [free text]

How old are you? [free text number]

Overall, which type of patch do your pupils mainly prefer to wear? (Patterned patch / plain patch / No preference)
In your experience overall, what best encourages your pupils to wear their patch? Please rank from most encouraging to least. Please move the options and place in order. (Force / Rewards / Desire to improve eyesight)
In your experience, which of the following limits your pupils' compliance with patching? Time it takes / People make fun/ It's painful or uncomfortable / It affects my friendships / It changes how I play with my friends / It stops me from learning in school
Do you think that computer games or apps that could see if you are wearing your patch and give you extra skins/power-ups/lives/rewards would make your pupils want to wear their patch? Yes / no

If so, how and why do you think this? [Free text]

Table S3: Questions to parents

Parents

Are you currently based in the UK? (Yes / no)

We want to reach as many people as possible with this survey. To help us, what are the first 2 letters of your postcode? [free text]

We want to reach as many people as possible with this survey. To help us, what country are you from? [free text]

How old are you? [free text]

Which of the following applies to you? (My child is currently having patch therapy / my child used to have patch therapy)
We are hoping to reach a wide range of voices with this survey, therefore we would like to know what is your highest educational attainment? [GCSE or equivalent, A Level, Graduate degree or higher]

Does your child find it more difficult to patch during the week or at weekends? (During the week / No difference / Weekends)

Overall does your child have a preferred patch type? (Patterned patch / plain patch / no preference)
In your experience overall, what best encourages your child to wear their patch? Please rank from most encouraging to least. Please move the options and place in order. (Force / Rewards / Desire to improve eyesight)
In your experience, which of the following limits your child's compliance with patching? (Time it takes / People make fun/ It's painful or uncomfortable / It affects my friendships / It changes how I play with my friends / It stops me from learning in school)
Do you think that computer games or apps that could see if your child was wearing their patch and give them extra skins/power-ups/lives/rewards would make your child want to wear their patch? (Yes / no)

Table S4: Questions for past patients

Ex-patients (over 18)

Are you currently based in the UK? (Yes / no)

We want to reach as many people as possible with this survey. To help us, what are the first 2 letters of your postcode? [free text]

We want to reach as many people as possible with this survey. To help us, what country are you from? [free text]

How old are you? [free text]
We are hoping to reach a wide range of voices with this survey, therefore we would like to know what is your highest educational attainment? (GCSE or equivalent, A Level, Graduate degree)

Overall, did you find it more difficult to patch during the week or at weekends? (During the week / No difference / Weekends)

Most of my patching was (Only at school / More school than home / Same at school and home / More home than school / Only at home)

Overall, did you have a preferred patch type? (Patterned patch, plain patch, no preference)
Overall, what best encouraged you to wear your patch? Please rank from most encouraging to least. Please move the options and place in order. (Force / Rewards / Desire to improve eyesight)
Overall in your experience, which of the following limited your compliance with patching? Please move these options and put them in order, with factors limiting compliance the most at the top. (Time it takes / People make fun/ It's painful or uncomfortable / It affects my friendships / It changes how I play with my friends / It stops me from learning in school)
Do you think that computer games or app that could see if the user was wearing their patch and give the user extra skins/power-ups/lives/rewards would have made you want to wear your patch?

Table S5: Questions to health professionals

Health professionals

Are you currently based in the UK? (yes / no)

We want to reach as many people as possible with this survey. To help us, what are the first 2 letters of your postcode? [free text]

We want to reach as many people as possible with this survey. To help us, what country are you from? [free text]

How old are you? [free text]

Overall, do your patients find it more difficult to patch during the week or at the weekends? (During the week / No difference / Weekends)
My patients spend most of their time patching: (Only at school / More school than home / Same at school and home / More home than school / Only at home)

Overall do your patients have a preferred patch type? (Patterned patch / plain patch / no difference
In your experience overall, what best encourages your patients to wear their patch? Please rank from most encouraging to least. Please move the options and place in order. (Force / Rewards / Desire to improve eyesight)

In your experience which of the following limits your patients' compliance with patching?
Do you think that computer games or apps that could see if you are wearing your patch and give you extra skins/power-ups/lives/rewards would make your patients want to wear their patch? (Time it takes / People make fun/ It's painful or uncomfortable / It affects my friendships / It changes how I play with my friends / It stops me from learning in school)
